# It doesn't matter what you say: FMRI correlates of voice learning and recognition independent of speech content

**DOI:** 10.1016/j.cortex.2017.06.005

**Published:** 2017-09

**Authors:** Romi Zäske, Bashar Awwad Shiekh Hasan, Pascal Belin

**Affiliations:** a Department of Otorhinolaryngology, Jena University Hospital, Jena, Germany; b Department for General Psychology and Cognitive Neuroscience, Institute of Psychology, Friedrich Schiller University of Jena, Jena, Germany; c Institute of Neuroscience, Newcastle University, Newcastle Upon Tyne, UK; d Aix Marseille Univ, CNRS, INT, Inst Neurosci Timone, Marseille, France; e Institute of Neuroscience and Psychology, University of Glasgow, Glasgow, Scotland, UK; f Département de Psychologie, Université de Montréal, Montréal, QC, Canada

**Keywords:** Voice memory, fMRI, Learning and recognition, Speech, TVA

## Abstract

Listeners can recognize newly learned voices from previously unheard utterances, suggesting the acquisition of high-level speech-invariant voice representations during learning. Using functional magnetic resonance imaging (fMRI) we investigated the anatomical basis underlying the acquisition of voice representations for unfamiliar speakers independent of speech, and their subsequent recognition among novel voices. Specifically, listeners studied voices of unfamiliar speakers uttering short sentences and subsequently classified studied and novel voices as “old” or “new” in a recognition test. To investigate “pure” voice learning, i.e., independent of sentence meaning, we presented German sentence stimuli to non-German speaking listeners. To disentangle stimulus-invariant and stimulus-dependent learning, during the test phase we contrasted a “same sentence” condition in which listeners heard speakers repeating the sentences from the preceding study phase, with a “different sentence” condition. Voice recognition performance was above chance in both conditions although, as expected, performance was higher for same than for different sentences. During study phases activity in the left inferior frontal gyrus (IFG) was related to subsequent voice recognition performance and same versus different sentence condition, suggesting an involvement of the left IFG in the interactive processing of speaker and speech information during learning. Importantly, at test reduced activation for voices correctly classified as “old” compared to “new” emerged in a network of brain areas including temporal voice areas (TVAs) of the right posterior superior temporal gyrus (pSTG), as well as the right inferior/middle frontal gyrus (IFG/MFG), the right medial frontal gyrus, and the left caudate. This effect of voice novelty did not interact with sentence condition, suggesting a role of temporal voice-selective areas and extra-temporal areas in the explicit recognition of learned voice identity, independent of speech content.

## 1 Introduction

In daily social interactions we easily recognize familiar people from their voices across various utterances (Skuk & Schweinberger, 2013). Importantly, listeners can recognize newly learned voices from previously unheard utterances suggesting the acquisition of high-level speech-invariant voice representations (Zäske, Volberg, Kovacs, & Schweinberger, 2014). Although it has been suggested that the processing of unfamiliar and familiar voices can be selectively impaired and relies on partially distinct cortical areas (Blank et al., 2014, Van Lancker and Kreiman, 1987, von Kriegstein and Giraud, 2004), the neural substrates underlying the transition from unfamiliar to familiar voices are elusive.

According to a recent meta-analysis (Blank et al., 2014) voice identity processing recruits predominantly right middle and anterior portions of the superior temporal sulcus/gyrus (STS/STG) and the inferior frontal gyrus (IFG). Specifically, functional magnetic resonance imaging (fMRI) research suggests that following low-level analysis in temporal primary auditory cortices, voices are structurally encoded and compared to long-term voice representations in bilateral temporal voice areas (TVAs) predominantly of the right STS (Belin et al., 2000, Pernet et al., 2015). This is in line with hierarchical models of voice processing (Belin et al., 2004, Belin et al., 2011). TVAs are thought to code acoustic-based voice information (Charest et al., 2013, Latinus et al., 2013) despite changes in speech (Belin & Zatorre, 2003), and irrespective of voice familiarity (Latinus et al., 2011, von Kriegstein and Giraud, 2004) and perceived identity (Andics, McQueen, & Petersson, 2013). The right inferior frontal cortex (IFC), by contrast, has been implicated in the perception of voice identity following learning irrespective of voice-acoustic properties (Andics et al., 2013, Latinus et al., 2011). This is in line with recent findings that the inferior prefrontal cortex is part of a broader network of voice-sensitive areas (Pernet et al., 2015). However, while previous studies have used various tasks and levels of voice familiarity to identify the neural correlates of voice identity processing, the neural mechanisms mediating the acquisition of high-level (invariant) voice representations during learning and subsequent recognition remain poorly explored.

Using a recognition memory paradigm we recently showed that voice learning results in substantial recognition of studied voices even when the test involved previously unheard utterances (Zäske et al., 2014). This supports the notion that listeners acquire relatively abstract voice representations (Belin et al., 2011) that allow for speaker recognition despite low-level variations between study and test, similar to findings in the face domain (Kaufmann et al., 2009, Yovel and Belin, 2013, Zimmermann and Eimer, 2013). Importantly, Zäske et al. (2014) found that study voices later remembered versus forgotten elicited a larger parietal positivity (∼250–1400 msec) in event-related potentials (ERPs). This difference due to memory (Dm) effect was independent of whether or not test speakers uttered the same sentence as during study and may thus reflect the acquisition of speech-invariant high-level voice representations. At test we observed OLD/NEW effects, i.e., a larger parietal positivity for old versus new voices (300–700 msec), only when test voices were recognized from the same sentence as heard during study. Crucially, an effect of voice learning *irrespective* of speech content was found in a reduction of beta band oscillations for old versus new voices (16–17 Hz, 290–370 msec) at central and right temporal sites. Thus, while the ERP OLD/NEW effect may reflect speech-dependent retrieval of specific voice samples from episodic memory, beta band modulations may reflect activation of speech-invariant identity representations. However, due to the lack of imaging data, the precise neural substrates of these effects are currently unknown.

By contrast, areas mediating the encoding and explicit retrieval of study items from episodic memory for various other stimulus domains. For instance, Dm effects, with stronger activation to study items subsequently remembered versus forgotten have been reported for words, visual scenes and objects including faces (Kelley et al., 1998, McDermott et al., 1999; reviewed in; Paller & Wagner, 2002), and musical sounds (Klostermann, Loui, & Shimamura, 2009). Essentially, this research suggests a role of inferior prefrontal and medial temporal regions for the successful encoding of visual items with laterality depending on the stimulus domain. For musical stimuli Dm effects were found in right superior temporal lobes, posterior parietal cortices and bilateral frontal regions (Klostermann et al., 2009). Similarly OLD/NEW effects for test items indicated successful retrieval of various visual and auditory stimuli (Klostermann et al., 2008, Klostermann et al., 2009, McDermott et al., 1999; reviewed in; Wagner, Shannon, Kahn, & Buckner, 2005). These studies suggest greater activation for correctly recognized studied versus novel items in parietal and/or prefrontal areas with stimulus-dependent laterality.

As from the above research it is unclear which brain areas might mediate learning and explicit recognition of voices we addressed this issue using fMRI. Specifically, we sought to disentangle speech-dependent and speech-invariant recognition by using either the same or a different sentence than presented at study in a recognition memory paradigm analogous to Zäske et al. (2014). Unlike previous fMRI research which focused on neural correlates of voice recognition for voices which were already familiar or have been familiarized prior to scanning (e.g., Latinus et al., 2011, Schall et al., 2015, von Kriegstein and Giraud, 2004) we investigate the brain responses during both learning of unfamiliar voices and recognition in a subsequent test. To use ecologically valid and complex speech stimuli, and yet to prevent interactive processing of the speaker's voice with the semantic content of speech, we presented German sentence stimuli to listeners who were unable to understand German. Specifically, the use of an unintelligible natural language should make it less likely for participants to engage extraneous top-down strategies (such as imagery) in the same sentence condition.

Based on the above research, a range of distributed brain areas may be involved in the learning and recognition of newly-learned voices. Based on literature on subsequent memory, we considered that during study, voices would differentially engage inferior prefrontal regions as well as temporal and posterior parietal regions depending on subsequent recognition performance (e.g., Kelley et al., 1998, Klostermann et al., 2009, McDermott et al., 1999). At test, studied compared to novel voices might increase parietal and/or (right) prefrontal areas, as suggested by research on newly-learned voices (Latinus et al., 2011) and research on OLD/NEW effects in episodic memory (Klostermann et al., 2008, Klostermann et al., 2009, McDermott et al., 1999; reviewed in; Wagner et al., 2005). Furthermore, studied compared to novel voices might decrease activity in (right) TVAs in line with findings on voice repetition (Belin & Zatorre, 2003). Specifically, while parietal OLD/NEW effects may be expected to be stimulus-dependent as these reflect episodic memory for a specific study item (Cabeza et al., 2012, Zäske et al., 2014), sensitivity to voice novelty in voice sensitive areas of the right TVAs and the IFC should be independent of speech content (Belin and Zatorre, 2003, Formisano et al., 2008, Latinus et al., 2011, Zäske et al., 2014).

## 2 Methods

### 2.1 Participants

Twenty-four student participants at the University of Glasgow, UK (12 female, all right-handed and unable to understand German, mean age = 21.6 yrs, range = 19–30 yrs) contributed data. None reported hearing problems, learning difficulties or prior familiarity with any of the voices used in the experiment. Data from two additional participants were excluded because one participant ended the scan prematurely and another understood German. Participants received a payment of £12. All gave written informed consent. The study was conducted in accordance with the Declaration of Helsinki, and was approved by the ethics committee of the College of Science and Engineering of the University of Glasgow.

### 2.2 Stimuli

Stimuli were voice recordings from 60 adult native speakers of German (30 female) aged 18–25 yrs (mean age = 21.9 yrs). Female speakers (mean age = 22.0 yrs) and male speakers (mean age = 21.9 yrs) did not significantly differ in age (*t*[58] = .231, *p* = .818). All speakers uttered 16 German sentences (8 of which started with the article “Der” and “Die”, respectively) resulting in 960 different stimuli. All sentences had the same syntactic structure and consisted of 7 or 8 syllables, e.g., “Der Fahrer lenkt den Wagen.” (The driver steers the car.), “Die Kundin kennt den Laden.” (The customer knows the shop.) cf. Supplementary material for transcripts and translations of German sentence stimuli. Speakers were asked to intonate sentences as emotionally neutral as possible. In order to standardize intonation and sentence duration and to keep regional accents to a minimum, speakers were encouraged to mimic as closely as possible a pre-recorded model speaker (first author) presented via loudspeakers. Each sentence was recorded 4–5 times in a quiet and semi-anechoic room by means of a Sennheiser MD 421-II microphone with a pop protection and a Zoom H4n audio interface (16-bit resolution, 48 kHz sampling rate, stereo). The best recordings were chosen as stimuli (no artifacts nor background noise, clear pronunciation). Using PRAAT software (Boersma & Weenink, 2001) voice recordings were cut to contain one sentence starting exactly at plosive onset of “Der”/“Die”. Voice recordings were then resampled to 44.1 kHz, converted to mono and RMS normalized to 70 dB. Mean sentence duration was 1,697 msec (SD = 175 msec, range 1,278–2,227 msec). Study and test voices were chosen based on distinctiveness ratings performed by an independent group of 12 German listeners (6 female, *M* = 22.4 yrs, range = 19–28 yrs). In this study, raters were presented with 64 voices (32 female) each uttering 5 sustained vowels (1.5 sec of the stable portion of [a:], [e:], [i:], [o:] and [u:]). They performed “voice in the crowd” distinctiveness ratings on a 6-point rating scale (1 = ‘non-distinctive’ to 6 = ’very distinctive’). In analogy to the “face in the crowd” task (e.g., Valentine and Bruce, 1986, Valentine and Ferrara, 1991) raters were instructed to imagine a crowded place with many people speaking simultaneously. Voices that would pop-out from the crowd should be considered distinctive. Sixty of those voices were used for the experiment. As voice distinctiveness affects voice recognition (Mullennix et al., 2011, Skuk and Schweinberger, 2013), we chose 6 female and 6 male study voices with intermediate levels of mean distinctiveness across vowels (i.e., values between the lower and upper quartile of the female and male distribution respectively).^2^ Mean distinctiveness did not differ between the female (*M* = 3.2, *SD* = .08) and the male (*M* = 3.2, *SD* = .07) study set (*t*[10] = −.080, *p* = .938). The remaining voices were used as test voices. As before distinctiveness did not differ between female (*M* = 3.2, *SD* = .29) and male (*M* = 3.3, *SD* = .25) test voices (*t*[46] = −.607, *p* = .547).

As practice stimuli, we used voices of 8 additional speakers (4 female) uttering 2 sentences not used in the main experiment. Stimuli were presented diotically via headphones (Sensimetrics-MRI-Compatible Insert Earphones, S14) with an approximate peak intensity of 65 dB(A) as determined with a Brüel & Kjær Precision Sound Level Meter Type 2206.

### 2.3 Procedure

**Fig. 1:**
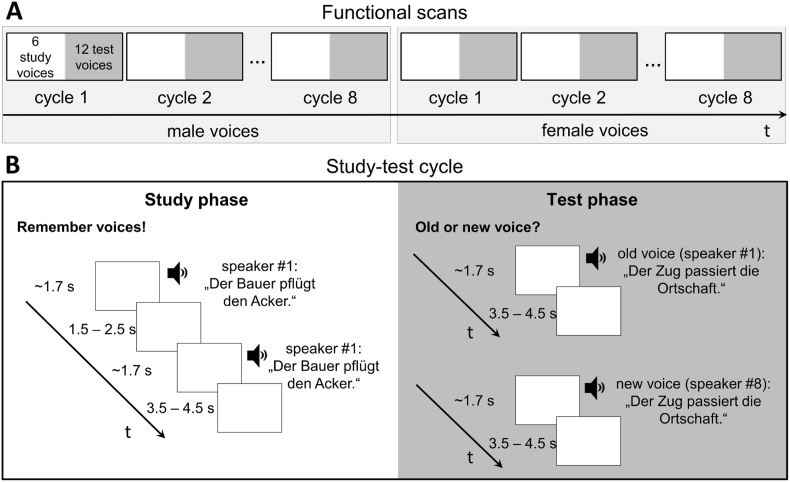
(A) Six male and six female study voices were presented in separate runs. Each run consisted of 8 study-test cycles in which study speakers were repeated and subsequently tested. At test, participants performed old/new classifications for 12 voices (6 old/6 new). Half of the old and new speakers repeated the sentence from the preceding study phase (same sentence condition), the other half uttered a different sentence (different sentence condition). (B) Trial procedure for one study-test cycle. During the study phase each speaker uttered the same sentence twice in succession. The example shows two trials for the “different sentence condition”: one with an “old” test voice and one with a “new” test voice. Study and test trials were presented in random order.

Participants were familiarized with the task outside the scanner by means of 4 study trials and 8 test trials for practice. Instructions were delivered orally according to a standardized protocol. For the main experiment and the subsequent voice localizer scan participants were asked to keep their eyes shut. The experiment was divided in two functional runs, one male and one female, in which participants learned 6 voices, respectively. Each run comprised 8 study-test cycles each consisting of a study phase with 6 voices and a subsequent test phase with 12 voices of the same sex, all presented in random order (cf. Fig. 1 for experimental procedures).

Scanning started with a silent interval of 60 sec before the beginning of the first study phase. Study phases were announced with a beep (500 msec) followed by 6 sec of silence. Participants were instructed to listen carefully to the 6 voices in order to recognize them in a subsequent test. In each of six study trials a different speaker was presented with two identical voice samples. The ISI between the two samples and between trials were jittered at 1.5–2.5 sec and 3.5–4.5 sec respectively. No responses were required on study trials.

Test phases were announced by two beeps (500 msec) followed by 6 sec of silence. Participants performed old/new classifications for the voices of 12 speaker identities. Six (old) test voices had been studied before and 6 (new) voices had not. For a given test phase, half of the test voices (3 old/3 new voices) said the same sentence as during the preceding study phase; the other half said a different sentence. Assignment of speakers to sentence conditions was randomly determined for each participant and remained constant throughout the experiment. Because each of two test sentences within a given test phase was always uttered by both “old” and “new” voices, sentence content could not serve as a cue for voice recognition. Sentence conditions (same/different) and test voice conditions (old/new) varied randomly between trials. Test trials consisted of one presentation of each test voice followed by a jittered ISI of 3.5s–4.5 sec within which responses were collected. Importantly, participants were instructed to respond as correctly as possible to *speaker identity*, i.e., regardless of sentence content, after voice offset. Responses were entered using the right index and middle finger placed on the upper two keys of a 4-key response box. Assignment of keys to “old” or “new” responses was counterbalanced between participants.

In order to improve learning the same set of 6 study voices was repeated across the 8 study-test cycles of each run, while new test voices were randomly chosen for each test phase among the 24 remaining speakers of the respective sex. With 24 speakers available for the 48 “new” test trials (6 new test voices for each of 8 test phases), “new” test voices were presented twice. Therefore, in order to minimize spurious recognition of “new” voices, these voices were never repeated within the same test phase and never with the same voice sample. After the first 8 cycles (first run) a new set of 6 study voices was used in the remaining 8 cycles (second run). With 16 sentences available overall, each run comprised a different set of 8 sentences. Voice gender and sentence sets (“Die”/”Der” sentences) were counterbalanced across participants. Assignment of study and test sentences to cycles varied randomly between participants. Notably, across all study-test cycles of each run the same 8 sentences were used both for the same and different sentence conditions respectively. Thus, overall phonetic variability of sentences was identical across both sentence conditions. This was to ensure that potential effects of sentence condition on voice learning could not be explained by differences in phonetic variability of the speech material (Bricker and Pruzansky, 1966, Pollack et al., 1954). Accordingly, while in the “same sentence condition” a given test sentence had always occurred in the directly preceding study phase, test sentences in the “different sentence condition” occurred as study sentences in another cycle of the respective run.

Taken together there were 96 study trials (2 runs × 8 cycles × 6 trials) and 192 test trials (2 runs × 8 cycles × 12 trials). Breaks of 20 sec were allowed after every cycle. In total, the experimental runs lasted about 50 min.

### 2.4 Image acquisition

Functional images covering the whole brain (field of view [FOV]: 192 mm, 31 slices, voxel size 3^3^ mm) were acquired on a 3-T Tim Trio Scanner (Siemens) using an echoplanar imaging (EPI) continuous sequence (interleaved, time repetition [TR]: 2.0 sec, multiecho [iPAT = 4] with 5 time echoes [TE]: 9.4 msec, 18.4 msec, 27.4 msec, 36.5 msec, and 45.5 msec, flip angle: 77°, matrix size: 64^2^). Two runs of ∼25 min (∼750 volumes) were acquired; 10 volumes were recorded with no stimulation at the end of a run to create a baseline. Between the two experimental runs, high-resolution T1-weighted images (anatomical scan) were obtained (FOV: 256 mm, 192 slices, voxel size: 1^3^ mm, flip angle: 9°, TR: 2.3 sec, TE: 2.96 msec, matrix size: 256^2^) for ∼10 min. After the second run, voice-selective areas were localized using a ‘‘voice localizer’’ scan in order to allow region of interest (ROI) analyses: 8 sec blocks of auditory stimuli containing either vocal or non-vocal sounds (Belin et al., 2000 – available online: http://vnl.psy.gla.ac.uk/resources_main.php); the voice localizer (FOV: 210 mm, 32 slices, voxel size: 3^3^, flip angle: 77°, TR: 2s, TE: 30 msec, matrix size: 70^2^).

### 2.5 Data analyses

#### 2.5.1 Behavioral data

Behavioral data were collapsed across the male and female runs and were submitted to analyses of variance (ANOVA) and *t*-tests using SPSS 19. Where appropriate, Epsilon corrections for heterogeneity of covariances (Huynh & Feldt, 1976) were performed throughout. Errors of omission were excluded (.9% of responses). We analyzed recognition performance in signal detection parameters (Stanislaw & Todorov, 1999) d-prime (d′) and response bias (C), as well as in accuracy data.

#### 2.5.2 FMRI data

Data were analyzed using SPM8 software (Welcome Department of Imaging Neuroscience; http://www.fil.ion.ucl.ac.uk/spm). During preprocessing anatomical images were aligned to the anterior and posterior commissure (AC–PC) and the orientation change was applied to all functional images, i.e., images from both the experimental runs and voice localizer scan. Functional scans were corrected for head motion by aligning all volumes of the five echo series to the first volume of the first run of the first echo series and, subsequently, to the mean volume. The anatomical scan (T1) was co-registered to the mean volume and segmented. Following the combination of the five echo series for the experimental runs, all functional scans (including voice localizer scan) and the T1 image were transformed to Montreal Neurological Institute (MNI) space using the parameters obtained from the segmentation. We kept the original voxel resolution of 1 mm^3^ for T1, and resampled the voxel resolution to 2^3^ mm for all functional scans (including voice localizer). All functional images were then spatially smoothed by applying a Gaussian kernel of 6 mm full-width at half mean (FWHM).

Functional data of the experimental runs were collapsed across the male and female runs and analyses were performed separately for study and test trials using the general linear model (GLM) implemented in SPM8. Each trial was treated as one single event in the design matrix. Thus, for study trials, one event consisted of two consecutive presentations of the same voice sample of a given speaker. For test trials, each event consisted of one voice sample of a given speaker. We performed both whole-brain and ROI-based analyses. A whole brain analysis is important as previous research suggested widespread candidate areas sensitive to voice learning and recognition (cf. Introduction). However, ROI-based analyses are still important to provide potential effects within TVAs.

First-level analyses for each participant involved the comparison (*t*-tests) of 14 conditions to the implicit baseline of SPM in order to determine cerebral regions which are more activated relative to baseline. Accordingly, study trials were sorted based on sentence condition (same sentence [same] *vs* different sentence [diff]) and subsequent recognition at test (hit *vs* miss) resulting in 4 conditions: same-hit, same-miss, diff-hit, and diff-miss. Test trials were sorted based on sentence condition (same *vs* diff) and voice recognition performance (hit, correct rejection [CR], miss and false alarm [FA]) resulting in 8 conditions: same-hit, same-CR, same-miss, same-FA, diff-hit, diff-CR, diff-miss, diff-FA. Trials with missing responses in study and test phases were sorted into 2 further conditions. Thus, the design matrix for the GLM contained a regressor for each of 14 conditions and 6 motion regressors.

Group-level ANOVAs were performed on individual contrasts across the brain volumes using three full (2 × 2) factorial designs: (1) Difference due to memory (Dm) effects were assessed for study trials in 2 sentence conditions (same/diff) × 2 subsequent recognition conditions (hit/miss), (2) OLD/NEW effects were assessed for *correct* test trials in 2 sentence conditions (same/diff) × 2 voice novelty conditions (old/new), and finally (3) errors were analyzed analogous to the second ANOVA, however including only *incorrect* test trials (2 sentence conditions [same/diff] × 2 voice novelty conditions [old/new]). Statistical significance was assessed at the peak level with a threshold of *p* < .001 (uncorrected) and with significant results reported for the cluster level at an extent threshold of 100 voxels, with *p* < .05 and Family-Wise Error (FWE) correction. Brain areas were identified using the xjView toolbox (http://www.alivelearn.net/xjview) and MNI-Talairach converter of Yale University website (http://sprout022.sprout.yale.edu/mni2tal/mni2tal.html). Brain maps were generated using MRIcron (http://people.cas.sc.edu/rorden/mricron/index.html).

For the TVA Localizer analysis, a univariate analysis was carried out as described in Belin et al. (2000). The design matrix for the GLM contained a voice and a non-voice regressor and 6 motion regressors. A contrast image of vocal versus non-vocal (*t*-test) was then generated per participant. Contrast images were then entered into a second-level random effects analysis (*p* < .05, FWE corrected). The resulting image was used as an explicit mask to the three group level ANOVAs described above.

## 3 Results

### 3.1 Performance

ANOVAs on signal detection parameters were performed with repeated measures on two sentence conditions (same/different) and four cycle pairs (1&2/3&4/5&6/7&8), in order to test the progression of learning throughout the experiment. For this analysis, 2 consecutive cycles were collapsed due to the otherwise low number of test trials per cycle (6 studied/6 novel voices per sentence condition, i.e., after merging male and female cycles). ANOVAs for accuracies were performed with the additional within-subjects factor voice novelty (old/new). Performance data are summarized in Table 1.

**Table 1:** Accuracies, sensitivity (d′), and response criteria (C) for sentence condition (same/diff), voice novelty condition (old/new), and cycle pairs with standard errors of the mean (SEM) in parentheses.

Sentence condition	Cycle pair	Old voices (Hits)	New voices (CR)	d’	C
Same	1_2	.79 (.02)	.43 (.04)	.68 (.11)	−.53 (.09)
	3_4	.79 (.02)	.46 (.04)	.73 (.13)	−.50 (.07)
	5_6	.76 (.03)	.47 (.04)	.69 (.12)	−.44 (.08)
	7_8	.78 (.02)	.54 (.04)	.92 (.13)	−.35 (.07)
Different	1_2	.54 (.04)	.55 (.03)	.23 (.11)	.02 (.08)
	3_4	.60 (.04)	.57 (.03)	.49 (.13)	−.04 (.08)
	5_6	.60 (.04)	.56 (.03)	.45 (.09)	−.06 (.08)
	7_8	.60 (.04)	.53 (.04)	.37 (.10)	−.10 (.10)

#### 3.1.1 Response criterion

Responses in the same sentence condition were more liberal than in the different sentence condition (*F*[1,23] = 20.16, *p* < .001, *η*_*p*_^2^ = .467). No further effects were observed.

#### 3.1.2 Sensitivity

**Fig. 2:**
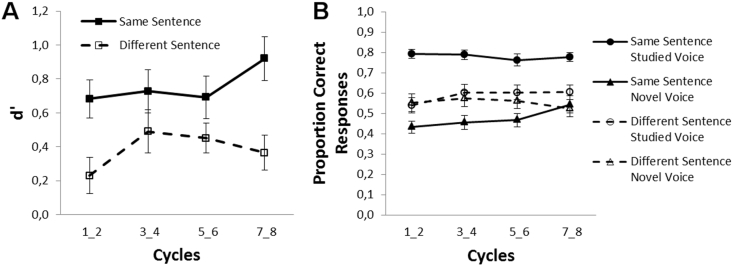
Voice recognition performance as reflected in (A) mean sensitivity d′ and (B) proportion correct responses depicted for sentence conditions and pairs of cycles (and voice novelty conditions). Error bars are standard errors of the mean (SEM).

We obtained higher d′ when voices were tested with the same sentence as in the study phase than with a different sentence (*F*[1,23] = 26.07, *p* < .001, *η*_*p*_^2^ = .531). Voice recognition performance was unaffected by cycle pairs (*F*[3,69] = 1.27, *p* = .293), but substantially above-chance (d′ > 0) in all conditions (3.48 < *t*s[23] < 6.94, *p*s ≤ .016, determined with one-sample *t*-tests, *p* Bonferroni-corrected for eight comparisons) but one condition: the *t*-test for voices presented with different sentences within the first cycle pair did not survive Bonferroni-correction, (*t*[23] = 2.12, *p* = .36), cf. Fig. 2(A).

#### 3.1.3 Accuracies

Significant main effects of sentence condition (*F*[1,23] = 20.40, *p* < .001, *η*_*p*_^2^ = .470) and voice novelty (*F*[1,23] = 18.37, *p* < .001, *η*_*p*_^2^ = .444) were qualified by interactions of sentence and voice novelty (*F*[1,23] = 20.32, *p* < .001, *η*_*p*_^2^ = .469) as well as of sentence condition, voice novelty and cycle pair (*F*[3,69] = 2.8, *p* = .047, *η*_*p*_^2^ = .108). Two separate ANOVAs for each voice novelty condition revealed a main effect of sentence condition both for old and new voices (*F*[1,23] = 38.41, *p* < .001, *η*_*p*_^2^ = .625 and *F*[1,23] = 5.25, *p* = .031, *η*_*p*_^2^ = .186, respectively). These effects reflected more correct responses to same sentences than to different sentences for old voices, and vice versa for new voices (see Fig. 2 B). The interaction of cycle pair and sentence was absent for old voices (*F*[3,69] = 1.59, *p* = .2, *η*_*p*_^2^ = .065) and reduced to a trend for new voices (*F*[3,69] = 2.47, *p* = .069, *η*_*p*_^2^ = .097), reflecting that the effect of sentence condition decreased with increasing number of cycles. When collapsed across cycles voice recognition scores were above chance (>.5) for old voices (*t*[23] = 14.71, *p* < .001 and *t*[23] = 2.77, *p* = .044 in the same sentence condition and different sentence condition, respectively), but not for new voices (*ps* > .05). All *p* values are Bonferroni-corrected.

### 3.2 FMRI results

#### 3.2.1 Whole-brain analysis

We calculated group-level, 2 × 2 factorial designs separately for 1) study trials, 2) correct test trials and 3) incorrect test trials (cf. Methods). For a summary of FMRI results please see Table 2. For study trials, we observed a significant main effect of sentence condition (same < diff) in the right fusiform gyrus (rFG; peak MNI coordinates [x y z] 42 −42 −22 mm^3^, *Z* = 4.64, cluster size: 225), cf. Supplementary Fig. 1 (Top) with smaller activation when the subsequent test sentence was the same as during study compared to a different sentence. Furthermore, we obtained an interaction of sentence condition and subsequent recognition in the left inferior frontal gyrus (left IFG, BA 47; peak MNI coordinates [x y z] −32 24 −6 mm^3^, *Z* = 4.36, cluster size: 247), cf. Supplementary Fig. 1 (Bottom). Post-hoc comparisons revealed that this was due to a Dm effect for voices presented in the same sentence condition with stronger activation for subsequent hits compared to misses. The reverse pattern was observed when speakers uttered a different sentence at test than at study.

**Table 2:** Coordinates of local maxima (MNI space in mm) for BOLD-responses in study and test phases as revealed by the whole brain analyses. Significant effects were significant on the peak level (*p* < .001 [uncorrected]) and for the respective clusters (*p* < .05 [FWE] as listed here) and are reported for an extent threshold of 100 voxels. Cluster size reflects the number of voxels per cluster.

Contrast	Cluster size	*p*	Z	x y z	Brain region

**ANOVA – Study trials (subsequent recognition)**					
sentence effect					
same > diff	n.s.				
same < diff	225	.005	4.64	42 −42 −22	right FG
subs. voice memory					
subs. hits > misses	n.s.				
subs. hits < misses	n.s.				
novelty × sentence	247	.003	4.36	−32 24 −6	left IFG
**ANOVA – Test trials (correct responses)**					
sentence effect					
same > diff	n.s.				
same < diff	n.s.				
voice novelty effect					
hits > CR	n.s.				
hits < CR	1225	<.001	4.76	52 20 26	right IFG/MFG
	250	.002	4.30	−16 14 8	left caudate
	563	<.001	4.17	66 −18 4	right STG^a^
	290	<.001	4.09	4 20 54	right area frontalis intermedia
novelty × sentence	138	.033	3.93	−38 2 2	left insula
**ANOVA – Test trials (incorrect responses)**					
sentence effect					
same > diff	n.s.				
same < diff	n.s.				
voice novelty effect					
misses > FA	n.s.				
misses < FA	n.s.				
novelty × sentence	n.s.				

a Note that this voice novelty effect (hits < CR) in the right pSTG was the only significant effect in the ROI analyses of the TVAs. ROI analyses were performed analogous to the whole brain analyses.

**Fig. 3:**
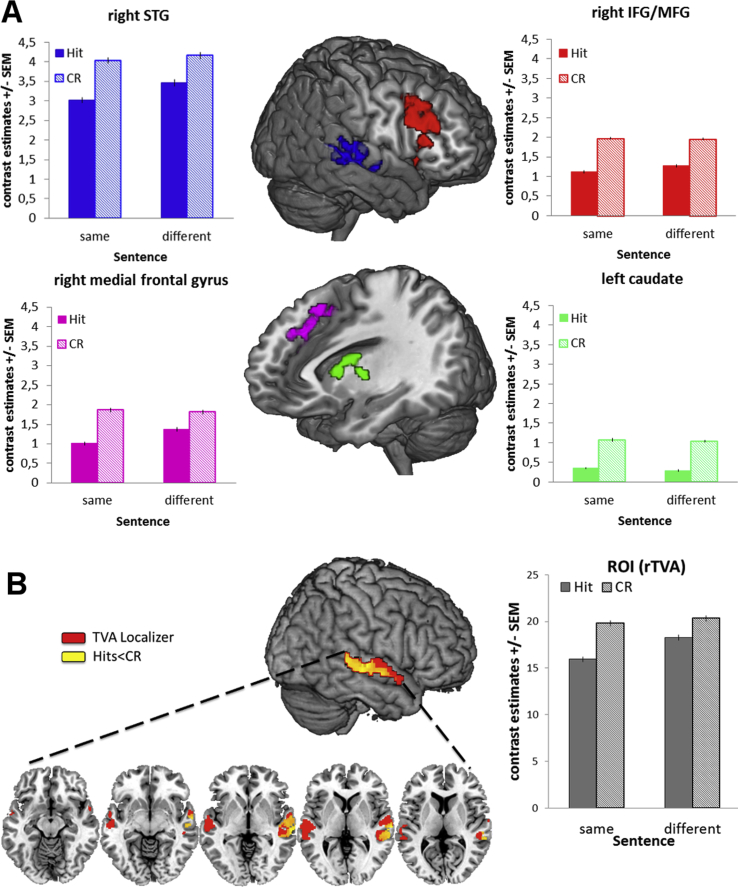
(A) Whole brain analysis of test phases. Brain areas sensitive to voice novelty (hits < CR) irrespective of sentence condition in the right STG, right IFG/MFG, right medial frontal gyrus, and the left caudate. (B) ROI analysis of test phases in bilateral voice-sensitive areas. Reduced activity to studied voices (hits) compared to novel voices (CR) independent of speech content were observed in the right STG with no effect of sentence condition.

For correct test trials, the 2 × 2 sentence (same/different) × voice novelty (hits/CR) factorial design yielded a main effect of voice novelty with significantly less activation for old voices correctly recognized (hits) compared to new voices correctly rejected (CR) in 4 different areas, cf. Fig. 3(A): (1) in a cluster of voxels of the right posterior superior temporal gyrus (pSTG; peak MNI coordinates 66 −18 4 [x y z], *Z* = 4.17, mm^3^ cluster size: 563); (2) the right inferior/middle frontal gyrus (rIFG/MFG, BA 22; peak MNI coordinates 52 20 26 [x y z], *Z* = 4.76, mm^3^ cluster size: 1225); (3) right medial frontal gyrus (BA8, peak MNI coordinates 4 20 54 [x y z], *Z* = 4.09, mm^3^ cluster size: 290); (4) left caudate (peak MNI coordinates −16 14 8 [x y z], *Z* = 4.30, mm^3^ cluster size: 250). These inverse OLD/NEW effects were independent of sentence condition. An interaction of sentence condition × voice novelty was observed in the left insula (peak MNI coordinates −38 2 2 [x y z], *Z* = 3.93, mm^3^ cluster size: 138), reflecting an OLD/NEW effect (hits > CR) in the same sentence condition, and the reverse pattern (hits < CR) in the different sentence condition, cf. Supplementary Fig. 2. For incorrect test trials, the 2 × 2 sentence (same/different) × voice novelty (miss/FA) factorial design yielded no significant main effects or interactions.

#### 3.2.2 TVAs – region of interest analyses

For ROI analyses in voice selective areas along bilateral STG, we conducted the same analyses as for the whole brain analyses. While there were no significant effects in the study phase or for test voices that had been incorrectly classified, test voices which had been correctly classified elicited smaller activity in the right TVAs when they had been previously studied (hits) compared to novel voices (CR), (BA22; peak MNI coordinates 66 −20 4 [x y z], *Z* = 4.88, mm^3^ cluster size: 406), cf. Fig. 3(B). This effect did not interact further with sentence condition.

## 4 Discussion

Here we report the first evidence that successful voice recognition following learning of unfamiliar voices engages a network of brain areas including right posterior temporal voice areas (TVAs), the right inferior/middle frontal gyrus (IFG/MFG) and medial frontal gyrus, as well as the left caudate nucleus. Furthermore, in the study phase we observed brain activity in the left IFG which was related to subsequent voice recognition performance.

### 4.1 Recognition performance

As a replication of earlier findings we show that voice learning with a few brief sentences results in above-chance voice recognition that generalizes to new speech samples (e.g., Legge et al., 1984, Sheffert et al., 2002, Zäske et al., 2014). This suggests that listeners have acquired voice representations which store idiosyncratic voice properties independent of speech content (see also Zäske et al., 2014). Notably, the present findings were obtained for listeners (mostly British) who were unfamiliar with the speakers' language (German). This is remarkable in light of research showing substantial impairments for the discrimination of unfamiliar speakers (Fleming, Giordano, Caldara, & Belin, 2014) and speaker identification following voice learning (Perrachione & Wong, 2007) for foreign versus native language samples of speech. Language familiarity effects likely arise from a lack of listeners' linguistic proficiency in the foreign language which impedes the use of phonetic idiosyncrasies for speaker identification. Note, however, that a direct comparison between studies is limited by the fact that discrimination of unfamiliar voices and the identification of individual speakers by name may invoke partly different cognitive mechanisms than the present task of old/new voice recognition (Hanley and Turner, 2000, Schweinberger et al., 1997a, Van Lancker and Kreiman, 1987).

Although above-chance voice recognition (d′) was achieved in both sentence conditions, performance was highest in the same sentence condition, i.e., when study samples were repeated at test. This is consistent with previous research (e.g., Schweinberger et al., 1997b, Zäske et al., 2014) and reflects some interdependence of speech and speaker perception (see also Perrachione and Wong, 2007, Perrachione et al., 2011, Remez et al., 2007). In terms of accuracies, however, the same sentence condition elicited both the highest and lowest performance, i.e., for old and for new voices, respectively. Accordingly, whenever test speakers repeated the study sentences, listeners tended to perceive their voices as old. This is also reflected in a more liberal response criterion in the same as compared to the different sentence condition. Note, that we obtained these results although it was pointed out to all participants prior to the experiment that sentence content was not a valid cue to speaker identity and that the task was voice recognition, not sentence recognition.

### 4.2 Neural correlates of voice recognition following learning

Our fMRI data revealed reduced activation for old voices correctly recognized as old compared to new voices correctly rejected as new in the right posterior TVAs as well as prefrontal and subcortical areas (right IFG/MFG and medial frontal gyrus as well as the left caudate nucleus). Crucially, these effects of voice novelty were unaffected by whether or not speakers repeated the study sentences at test suggesting that activity in these areas is related to genuine voice identity processing, i.e., *independent* of low-level speech-based acoustic variability. This finding parallels our recent report of electrophysiological correlates of voice recognition independent of speech content (Zäske et al., 2014). Essentially, Zäske and colleagues showed that successful voice recognition independent of speech was accompanied by a reduction of beta band oscillations (16–17 Hz, 290–370 msec) for old versus new test voices at central and right temporal sites. Note that this right-lateralized topography of ERP recognition effects is overall in line with the present finding of predominantly right-hemispheric involvement in voice recognition. However, although Zäske et al. used the same task and an almost identical design and stimulus set, the main difference is that in the present study, we investigated foreign-language voice recognition rather than native-language voice recognition. While the underlying neural processes may therefore not be completely comparable (Perrachione, Pierrehumbert, & Wong, 2009), it remains possible that the electrophysiological voice recognition effect (Zäske et al., 2014) and the present effect in BOLD responses are related to a similar mechanism. Specifically, we suggest that the reduction in activity in the above network of brain areas reflects access to speech-independent high-level voice representations acquired during learning.

With respect to the TVA and the rIFC, our findings converge well with previous reports that both are voice sensitive areas (Belin et al., 2000, Blank et al., 2014) which respond to acoustic voice properties and perceived identity information, respectively (Andics et al., 2013, Latinus et al., 2011). Furthermore, the rIFC has been associated with the processing of vocal attractiveness (Bestelmeyer et al., 2012) and emotional prosody (Fruehholz, Ceravolo, & Grandjean, 2012) as well as the representation of voice gender (Charest et al., 2013). Although there is no consensus as yet on the exact function of the rIFC in voice processing, two recent studies suggest that it codes perceived identity of voices in a prototype-referenced manner independently of voice-acoustic properties (Andics et al., 2013, Latinus et al., 2011). Specifically, Andics et al. (2013) showed that repeating prototypical versus less prototypical voice samples of newly-learned speakers leads to an adaptation-induced reduction of activity in the rIFC. Based on the above studies, the present response reduction could in part reflect neural adaptation to old voices relative to new voices.

Alternatively, the present effects may be related to the *explicit* recognition of studied voices. To consider this possibility, we have analyzed incorrect trials analogous to correct trials. Specifically, we reasoned that if voice novelty modulates activity in the same or in overlapping brain areas for both types of trials, this may be indicative of implicit repetition-related effects, rather than explicit recognition. Since no significant effects emerged from these analyses we therefore favor the view that the present novelty effects reflect explicit recognition of learned voice identity. This would be in line also with neuroimaging research demonstrating that explicit recognition of a target voice among other voices activates bilateral frontal cortices compared to a task requiring the recognition of speech content (von Kriegstein & Giraud, 2004). In that study, bilateral frontal cortices were less activated during attention to voices of (personally) familiar speakers compared to unfamiliar speakers. This is similar to the present study where with increasing voice familiarity, responsiveness of the rIFG/MFG decreases (old < new voices). Additionally, von Kriegstein and Giraud showed that TVAs in the right STS functionally interacted with the right inferior parietal cortex and the right dorsolateral prefrontal cortex during the recognition of unfamiliar voices. The latter finding was attributed to increased difficulty of recognizing unfamiliar voices. Note, however, that unfamiliar voices in that study were not completely unfamiliar. Instead, and similar to the present study, von Kriegstein and Giraud had briefly familiarized participants with all voices and sentences prior to the testing session. Therefore, rather than indicating task difficulty, functional connections of TVAs with prefrontal cortex in that study may alternatively reflect explicit recognition of newly-learned voice identities, similar to the present study.

In addition, voice novelty was also found to modulate activity in the right medial frontal gyrus (BA8) and the left caudate nucleus. Although BA8 has been related to a number of cognitive functions, in the context of the present study, reduced responses in this area for old compared to new voices could reflect relatively higher response certainty (Volz, Schubotz, & von Cramon, 2005) for studied voices. Caudate nuclei have been suggested to mediate stimulus-response learning (Seger & Cincotta, 2005) and response inhibition (Aron et al., 2003). Accordingly, the present modulation in this area may be related to response selection processes during voice classification.

At variance with previous research on episodic memory for other classes of stimuli (reviewed in Cabeza et al., 2012, Cabeza et al., 2008), we did not find classical OLD/NEW effects (hits > CR) for voices in parietal cortex areas. This may be due to the present analysis approach which either targeted the whole brain with low statistical power, or regions of interest (TVAs) outside the parietal lobe.

Interestingly, the left insula was also sensitive to voice novelty, however, with voice novelty effects depending on sentence condition. When speakers repeated the study sentences at test, old voices enhanced left insula activity relative to new voices. The reverse pattern emerged when test speakers uttered a different sentence. The insula has been implicated in many tasks and has been discussed as a general neural correlate of awareness (reviewed in Craig, 2009). In the context of auditory research, the right insula has been suggested to play a role in the processing of conspecific communication sounds in primates (Remedios, Logothetis, & Kayser, 2009). In humans, the left insula has been associated with the processing of pitch patterns in speech (Wong, Parsons, Martinez, & Diehl, 2004) and motor planning of speech (Dronkers, 1996). It is further sensitive to non-linguistic vocal information including emotional expressions (Morris, Scott, & Dolan, 1999) and voice naturalness (Tamura, Kuriki, & Nakano, 2015). In general, the insulae have been found to respond more strongly to stimuli of negative valence (reviewed in Phillips, Drevets, Rauch, & Lane, 2003) including negative affective voices (Ethofer et al., 2009). Furthermore, previous research suggest that insula activity may reflect subjective familiarity with stronger responses in a network of brain areas including the insular cortex for (perceived as) new stimuli compared to familiar or repeated stimuli (e.g., Downar et al., 2002, Linden et al., 1999, Plailly et al., 2007).

In the present study, the strongest responses in the left insula have emerged for test samples that were either identical to studied voice samples, i.e., old voices which repeated the study sentence at test, or which were maximally different from the studied samples, i.e., new voices uttering a different sentence at test. Based on the above research one could speculate that two mechanisms underlie the present activity pattern: while the recognition of stimulus-specific prosody in the same sentence condition may have enhanced insula activity for old relative to new voices, a particularly pronounced feeling of “unfamiliarity” for different test sentences when uttered by new voices relative to old voices may have increased insula activity.

### 4.3 Neural correlates of subsequent voice memory

Here we show for the first time that activity in the left IFG (BA47) interacts with subsequent voice memory, thereby extending the episodic memory literature by an important new class of auditory stimuli. Interestingly, the Dm effect for voice memory depended on sentence condition: 1) study voices subsequently remembered elicited stronger responses than study voices subsequently forgotten when speakers uttered the same sentences at study and at test (classic Dm); 2) conversely, voices subsequently remembered elicited weaker responses than study voices subsequently forgotten when speakers uttered different sentences at study and at test (inverse Dm). The first finding is in line with previous reports of Dm effects for various stimuli as observed in a network of areas including left and/or right inferior prefrontal regions for identical study and test items (e.g., Kelley et al., 1998, Klostermann et al., 2009, McDermott et al., 1999, Ranganath et al., 2003).

The second effect, i.e., inverse Dm, has previously been related to unsuccessful encoding of study items into memory, however with effects typically located in ventral parietal and posteromedial cortex (Cabeza et al., 2012, Huijbers et al., 2013) rather than in the IFG. This inconsistency may be resolved when considering that the present effects may reflect combined effects of voice encoding and semantic retrieval processes. The left IFG, and BA47 in particular, has been repeatedly associated with language processing (e.g., Demb et al., 1995, Lehtonen et al., 2005, Sahin et al., 2006, Wong et al., 2002). For instance, Wong and colleagues observed activations in the left BA47 in response to backward speech, but not for meaningful forward speech suggesting that this area is involved in the effortful attempt to retrieve semantic information in (meaningless) backward speech. Similarly, although our participants were unable to understand the semantic content of the German utterances, the present Dm effects may reflect the attempt to nevertheless assign meaning to unintelligible speech. Depending on sentence condition, this process might have elicited different response patterns in the left IFG: while the association of semantic meaning with study voices provided a beneficial retrieval cue for the same stimuli at test (classic Dm), it may have compromised voice recognition from different test sentences (inverse Dm) which had not been previously associated with the speaker.

An unexpected finding was that responses in the right fusiform gyrus (FG) were decreased when study voices were subsequently tested with the same sentence relative to different sentences. The right FG is part of the face perception network and hosts the fusiform face area (FFA; Kanwisher, McDermott, & Chun, 1997). Although functional and anatomical coupling of the FFA and TVA have been reported for familiar speakers (Blank et al., 2011, von Kriegstein et al., 2005), it is difficult to reconcile these findings with the present sentence effect. As a possible mechanism, learning unfamiliar voices may have triggered facial imagery as mediated by the right FG. However, it remains to be explored why this effect was stronger in the same compared to the different sentence condition.

## 5 Conclusions

In conclusion, the present study reports brain areas involved in the learning and recognition of unfamiliar voices. This relatively widespread network may serve several sub-functions: During voice learning brain activity in the left IFG was related to subsequent voice recognition performance which further interacted with speech content. This suggests that the left IFG mediates the interactive processing of speaker and speech information while new voice representations are being built. During voice recognition, correct recognition of studied compared to novel voices was associated with decreased activation in voice-selective areas of the right pSTG and IFG/MFG, medial frontal gyrus, as well as the left caudate nucleus. Importantly, these effects were independent of speech content. We therefore suggest that these areas subserve the access to speech-invariant high-level voice representations for successful voice recognition following learning. Specifically, while the right pSTG and IFG/MFG may process idiosyncratic information about voice identity, the medial frontal gyrus and left caudate may be involved in more general mechanisms related to response certainty and response selection.

In view of other research pointing to differential voice processing depending on whether listeners are familiar with the speakers' language (Perrachione and Wong, 2007, Perrachione et al., 2009), the precise role of comprehensible speech for neuroimaging correlates of voice learning will be an interesting question for future research. Since we obtained the present findings with listeners who were unfamiliar with the speaker's language, the present findings arguably reflect a rather general mechanism of voice learning that is largely devoid of speech-related semantic processes.

## Footnotes

2 Voice selection was based on vowel stimuli. However, for the main experiment we used sentence stimuli assuming that the perception of voice distinctiveness should be correlated between different samples of speech.
