# Supplementary material for: It doesn't matter what you say: FMRI correlates of voice learning and recognition independent of speech content
Source: Cortex. 2017 Sep;94:100–12. doi: 10.1016/j.cortex.2017.06.005 (PMC5576914; doi:10.1016/j.cortex.2017.06.005)
Supplement: Supplementary file 1 [file mmc1.docx]

Supplementary Material

Figures

*
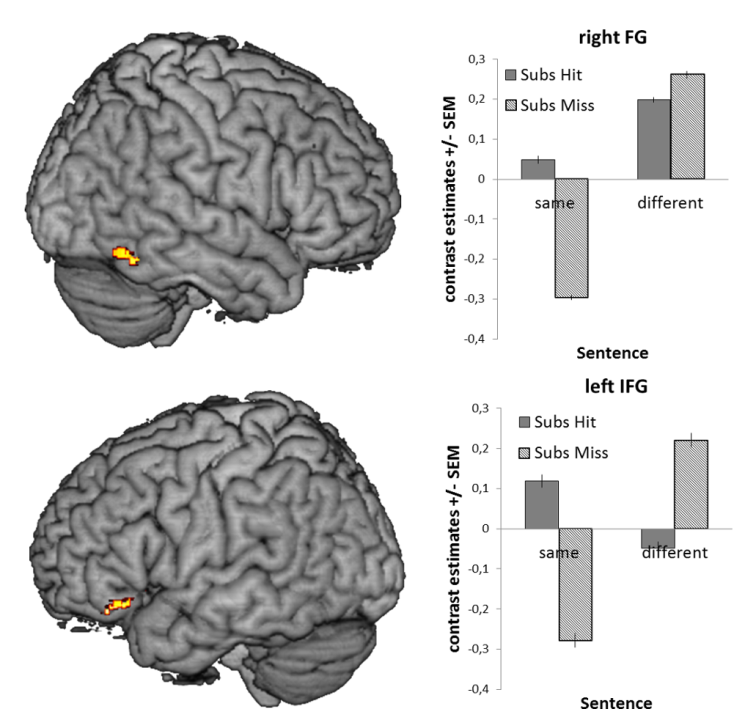
Supplementary Fig. 1.* Whole brain analysis of study phases. Top: The right fusiform gyrus was sensitive to sentence condition (same < different) irrespective of subsequent voice recognition. Bottom: Activity in the left inferior frontal gyrus (left IFG) was modulated by the interactive effect of sentence condition and subsequent voice recognition. This was due to Dm effects (subsequent hits > misses) when speakers uttered the same sentences at study as at test, and the reverse pattern (subsequent hits < misses) when test speakers uttered a different sentence than heard during study.

*
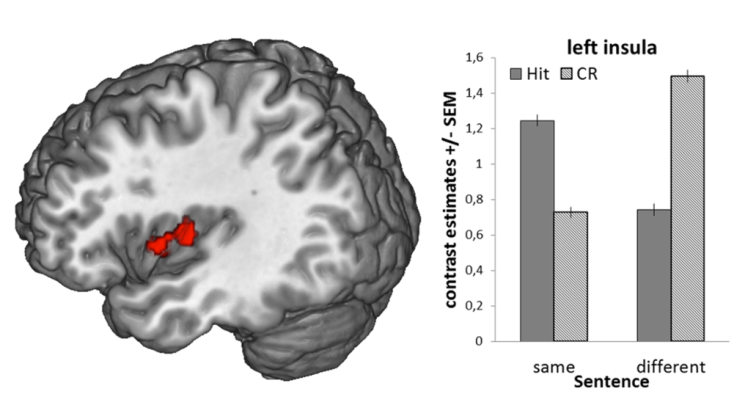
*

*Supplementary Fig. 2.* Whole brain analysis of test phases. The left insula was sensitive to the interaction of sentence condition and voice novelty. This was due to an OLD/NEW effect (hits > CR) in the same sentence condition, and the reverse pattern (hits < CR) in the different sentence condition.

Transcripts of German sentence stimuli (and practice stimuli) with their English translations

Der Autor schreibt das Kapitel. The author writes the chapter.

Der Bauer pflügt den Acker. The farmer ploughs the field.

Der Fahrer lenkt den Wagen. The driver steers the car.

Der Hase frisst die Möhre. The hare eats the carrot.

Der Hund vergräbt den Knochen. The dog buries the bone.

Der Maler streicht die Wohnung. The painter paints the flat.

Der Richter verliest das Urteil. The judge reads the verdict.

Der Zug passiert die Ortschaft. The train passes the town.

(Der Lehrer erhält die Nachricht.) (The teacher receives the message.)

Die Ärztin ruft die Schwester. The doctor calls the nurse.

Die Frau bügelt die Wäsche. The woman irons the clothes.

Die Fremde beherrscht die Lage. The stranger controls the situation.

Die Katze durchquert den Garten. The cat walks across the garden.

Die Kundin kennt den Laden. The customer knows the shop.

Die Mutter wischt den Boden. The mother cleans the floor.

Die Nachricht erreicht die Stadt. The news reaches the town.

Die Postfrau bringt die Zeitung. The postwoman delivers the newspaper.

(Die Nachfrage bestimmt den Preis.) (The demand determines the price.)
